# Dapoxetine and premature ejaculation

**DOI:** 10.1590/S1677-5538.IBJU.2023.9908

**Published:** 2023-08-07

**Affiliations:** 1 Hospital Federal da Lagoa Serviço de Urologia Rio de Janeiro RJ Brasil Serviço de Urologia, Hospital Federal da Lagoa, Rio de Janeiro, RJ, Brasil; 2 Universidade do Estado do Rio de Janeiro Unidade de Pesquisa Urogenital Rio de Janeiro RJ Brasil Unidade de Pesquisa Urogenital - Universidade do Estado do Rio de Janeiro - Uerj, Rio de Janeiro, RJ, Brasil

## COMMENT

## EJACULATORY PHYSIOLOGY

The ejaculatory reflex comprises a complex reflex synchrony involving the central and peripheral nervous systems. Receptors and sensory areas, afferent pathways, cerebral sensory areas, cerebral motor centers, spinal motor centers, and efferent motor centers are involved in the ejaculatory reflex. These areas also jointly modulate other aspects of sexual response (1).

Neurochemically, this reflex involves a complex interaction between serotonergic and dopaminergic and, secondarily, cholinergic, adrenergic, oxytocinergic, and γ-aminobutyric (GABA) neurons. While dopamine promotes seminal emission/ejaculation via D2 receptors, serotonin is inhibitory. This aspect needs to be clear, as knowledge of this neurophysiology will guide the treatment: serotonergic stimulation of 5-HT2c receptors results in delayed ejaculation, while stimulation of postsynaptic 5-HT1a receptors results in shortening of ejaculatory latency (2–4).

The peripheral events leading to ejaculation are controlled by the synergistic activation of autonomic functions (sympathetic and parasympathetic) and somatic portions of the nervous system. The origin of the autonomic and somatic motor efferent roots originates in the thoracolumbar and lumbosacral segments. Autonomic and somatic coordination are controlled in the L3–L5 region by a group of neurons called spinal ejaculatory generators. This nucleus, in conjunction with the autonomic system and the spinal somatic nuclei, is capable of stimulating or inhibiting the entry of genital and supraspinal sensory stimuli.

In higher centers, specific areas are activated during ejaculation, such as the bed nucleus of the stria terminalis, the amygdala, the preoptic nucleus, and the thalamus.

We prepared a didactic scheme that summarizes the aforementioned aspects:

## EJACULATORY REFLEX

After this review of neurophysiology, we can move on to deepening the ejaculatory process. To facilitate understanding, it is subdivided into three synchronized phases: emission, ejection (or penile expulsion) and orgasm.

Emission consists of contractions of the vas deferens and seminal vesicles, with the expulsion of sperm and seminal fluid into the prostatic urethra. This step is mediated by synergistic sympathetic and parasympathetic activation (T10-L2). The ejection phase is mediated by a spinal sympathetic reflex and somatic nerves (S2 to S4) from the pudendal nerve and involves pulsatile contractions of the bulbospongiosus, ischiocavernosus, and levator ani muscles along with relaxation of the external urinary sphincter. The internal urinary sphincter (bladder neck) closes to prevent retrograde flow into the bladder. The external urinary sphincter also presents cyclic contractions during semen ejection, helping in its expulsion.

Ultimately, orgasm is the result of brain processing of sensory stimuli from the pudendal nerve by increased pressure in the posterior urethra, sensory stimuli from the verumontanum, and contraction of the bulbourethral and sexual organs.

## SELECTIVE SEROTONIN REUPTAKE INHIBITORS (SSRIs)

These are a class of drugs prescribed for the treatment of a variety of mood disorders, such as depression. O use of SSRIs to treat PE is based on the observation that delayed ejaculation and anorgasmia are common side effects of this class of drugs used for a long time off-label. SSRIs, alone at low doses or in combination with psychosexual counseling, are widely accepted as first-line treatments for lifelong PE and are recommended by leading urology guidelines.

**Figure 1:**
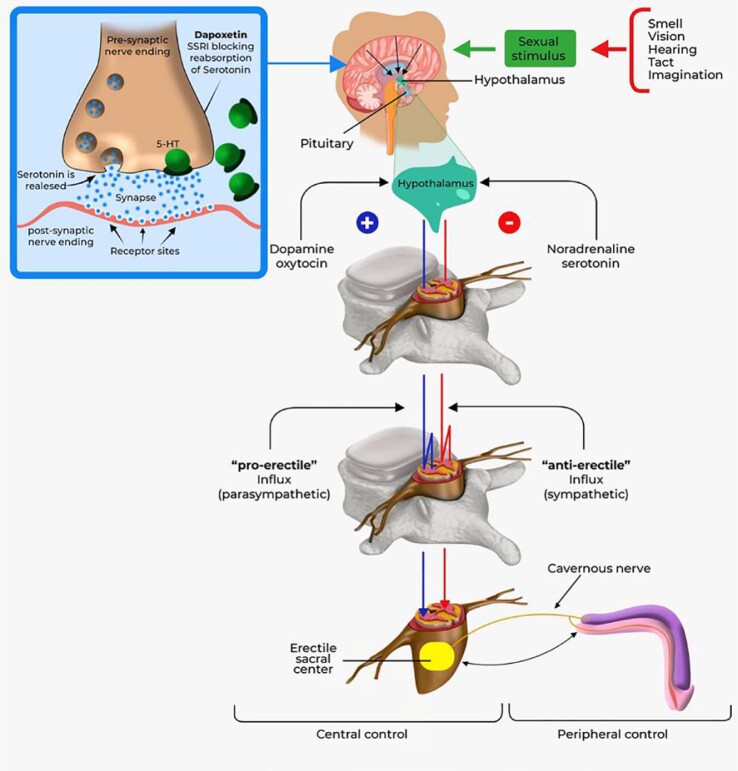
Ejaculatory physiology and mechanism of action of dapoxetine and other serotonin reuptake inhibitors.

Men with acquired PE usually receive targeted therapy with the aim of resolving the underlying etiology of your PE, with or without the addition of SSRIs, depending on clinical judgment. SSRIs act to block the axonal reuptake of serotonin from the synaptic cleft of central serotonergic neurons by 5-HT transporters, which desensitize 5-HT1A and 5-HT1B receptors (Figure-1) (5, 6).

Although ejaculation delay can be observed within a few days, it is necessary to administer the drugs for at least 2-3 weeks to maximize their therapeutic effects. Most SSRIs have been shown to delay ejaculatory time by increasing the average IELT by up to 13.2 times. However, chronic use of these drugs can increase the likelihood of unwanted adverse events such as decreased libido, anorgasmia and impotence/erectile dysfunction, and their sudden discontinuation can lead to SSRI withdrawal syndrome, which can last for more than a week and be accompanied even by suicidal ideation. Therefore, caution is required in handling these medications, starting use gradually as well as gradually weaning. Following this reasoning, the ideal SSRI should have a rapid onset of action and elimination, good tolerability, fewer adverse effects and be formulated for use as an on-demand treatment. And here we come to the high point of this editorial: dapoxetine is a short-acting SSRI option that meets the above PE treatment requirements.

## DAPOXETINE

Dapoxetine, or dapoxetine hydrochloride (N-dimethyl-(a)-[2-(1-naphthalenyloxy)ethyl]-benzenemethanamine) has a structure similar to fluoxetine and works similarly to other SSRIs, inhibiting the transporter that performs serotonin reuptake in the pre-synaptic cleft, whith minimal effects on norepinephrine and dopamine reuptake receptors. It is rapidly absorbed after administration but may change absorption in the presence of other foods. Its elimination is biphasic, with an initial half-life of approximately 1.3-1.4 hours and a terminal half-life of 21 hours, depending on the dose. Compared to longer-acting SSRIs such as fluoxetine and paroxetine, dapoxetine reaches steady-state plasma concentrations within four days due to its short half-life, being metabolized in the liver by cytochrome P450 isoenzymes and excreted in the urine without any effects on the enzymes of cytochrome P450 (7).

It is worth mentioning that for patients who present an anxious component, dapoxetine used on demand will not treat generalized anxiety, and its indication should be made in accordance with the observation of this clinical aspect. For this profile of patients, exclusive use on demand will not bring benefits to other areas of life, and the very use of dapoxetine with the aim of improvement in the sexual sphere, due to the basic anxious character, can induce a state of anxiety regarding an expected response in the improvement of the ejaculatory pattern.

It is also important to point out that in the case of choosing to replace a long-acting medication (SSRI) with dapoxetine, adequate weaning over the course of weeks should be carried out, not simply replacing the drugs.

Finally, as a conclusion, we observed that dapoxetine is a drug that fills an important space in the management of premature ejaculation, offering a safer and more comfortable treatment, mainly for the urologist, through the administration of an on-demand central nervous system action drug.
